# Prediction of prolonged length of stay on the intensive care unit in severely injured patients—a registry-based multivariable analysis

**DOI:** 10.3389/fmed.2024.1358205

**Published:** 2024-06-05

**Authors:** Rolf Lefering, Christian Waydhas, TraumaRegister DGU

**Affiliations:** ^1^Institute for Research in Operative Medicine, Faculty of Health, University Witten/Herdecke, Cologne, Germany; ^2^Department of Trauma Surgery, University Hospital Essen, University Duisburg-Essen, Essen, Germany

**Keywords:** trauma and injuries, intensive care, length of stay, registries trauma and injuries, registry, prediction models

## Abstract

**Purpose:**

Mortality is the primary outcome measure in severely injured trauma victims. However, quality indicators for survivors are rare. We aimed to develop and validate an outcome measure based on length of stay on the intensive care unit (ICU).

**Methods:**

The TraumaRegister DGU of the German Trauma Society (DGU) was used to identify 108,178 surviving patients with serious injuries who required treatment on ICU (2014–2018). In a first step, need for prolonged ICU stay, defined as 8 or more days, was predicted. In a second step, length of stay was estimated in patients with a prolonged stay. Data from the same trauma registry (2019–2022, *n* = 72,062) were used to validate the models derived with logistic and linear regression analysis.

**Results:**

The mean age was 50 years, 70% were males, and the average Injury Severity Score was 16.2 points. Average/median length of stay on ICU was 6.3/2 days, where 78% were discharged from ICU within the first 7 days. Prediction of need for a prolonged ICU stay revealed 15 predictors among which injury severity (worst Abbreviated Injury Scale severity level), need for intubation, and pre-trauma condition were the most important ones. The area under the receiver operating characteristic curve was 0.903 (95% confidence interval 0.900–0.905). Length of stay prediction in those with a prolonged ICU stay identified the need for ventilation and the number of injuries as the most important factors. Pearson’s correlation of observed and predicted length of stay was 0.613. Validation results were satisfactory for both estimates.

**Conclusion:**

Length of stay on ICU is a suitable outcome measure in surviving patients after severe trauma if adjusted for severity. The risk of needing prolonged ICU care could be calculated in all patients, and observed vs. predicted rates could be used in quality assessment similar to mortality prediction. Length of stay prediction in those who require a prolonged stay is feasible and allows for further benchmarking.

## Introduction

Most initiatives for quality assessment of the treatment of severely injured patients focus on mortality as primary outcome. This is reasonable since mortality rates range from 5% to 20% depending on the inclusion criteria. The German TraumaRegister DGU® (TR-DGU) of the German Trauma Society (DGU) considers reduction of hospital mortality as its primary aim as well. This registry includes severely injured patients admitted to hospital with trauma team activation who needed intensive care, or died. Specific scoring and prediction systems have been developed and validated to estimate the risk of death [RISC (1) and RISC II (2)]. Participating hospitals receive annual quality reports where observed and predicted mortality are compared.

However, for surviving patients only process parameter have been implemented as quality indicators (3). Length of Stay (LoS) in hospital or on the intensive care unit (ICU) could well be considered as a relevant outcome measure in survivors (4). A shorter length of stay would also be preferable from an economic point of view. But an unadjusted comparison of LoS data across hospitals would be misleading since LoS depends on several factors. Usually, a more severely injured patient would require a more intense therapy, and sometimes repeated operations, associated with a longer LoS (5). There are also patient-related factors with an effect on LoS, like age or concomitant diseases, especially in the elderly. Finally, also complications like (multiple) organ failure, or sepsis, determine the required LoS. For example, Böhmer et al. found that, after adjustment, a sepsis would prolong the ICU stay by 8 days, and organ failure would prolong ICU stay by 2–8 days on average, depending on the failing organs (5).

The present analysis aims to predict LoS on ICU as a means of benchmarking hospital treatment. However, LoS is not easy to predict since LoS data are rather skewed with a large number of patients requiring a short stay only, and a much smaller number of cases with a rather long need for intensive care. This small group of patients who require a prolonged LoS on ICU consume a considerable amount of resources (6). Several models to predict LoS on ICU exist already (7), but they focus on all cases and not just on prolonged ICU stay, or consider a mixed ICU population, or they did not include relevant predictors specifically for trauma patients available in our registry. According to Kramer et al., we followed a two-step approach to LoS prediction in survivors (6): In a first step we aimed to predict the probability for a prolonged ICU stay, and in a second step ICU LoS was sought to be predicted in those patients requiring a prolonged stay.

## Methods

This is a retrospective analysis of existing registry data from surviving patients with severe injuries The derived models were validated with contemporary data from the same registry, imitating the application of these models.

### TraumaRegister DGU®

The TraumaRegister DGU® (TR-DGU) of the German Trauma Society (Deutsche Gesellschaft für Unfallchirurgie, DGU) was founded in 1993. The aim of this multi-center database is a pseudonymized and standardized documentation of severely injured patients.

Data are collected prospectively in four consecutive time phases from the site of the accident until discharge from hospital: (A) Pre-hospital phase, (B) Emergency room and initial surgery, (C) Intensive care unit and (D) Discharge. The documentation includes detailed information on demographics, injury pattern, comorbidities, pre- and in-hospital management, course on the intensive care unit (ICU), relevant laboratory findings including data on transfusion, and outcome of each individual. The inclusion criterion is admission to hospital via the emergency room (trauma team activation) with subsequent intensive or intermediate care. Patients who reached the hospital with vital signs but died before admission to ICU were included as well.

The infrastructure for documentation, data management, and data analysis is provided by AUC—Academy for Trauma Surgery (AUC—Akademie der Unfallchirurgie GmbH), a company affiliated to the German Trauma Society. The scientific leadership is provided by the Committee on Emergency Medicine, Intensive Care and Trauma Management (Sektion NIS) of the German Trauma Society. The participating hospitals submit their data pseudonymised into a central database via a web-based application. Scientific data analysis is approved according to a peer review procedure laid down in the publication guideline of TR-DGU.

The participating hospitals are primarily located in Germany (90%), but a rising number of hospitals of other countries contribute data as well (presently Austria, Belgium, Finland, Luxembourg, Slovenia, Switzerland, The Netherlands, and the United Arab Emirates). Currently, approx. 30,000 cases from over 650 hospitals are entered into the database per year. Participation in TR-DGU is voluntary. For hospitals associated with TraumaNetzwerk DGU®, however, the entry of at least a basic data set is mandatory for reasons of quality assurance.

This study was conducted according to the publication guideline of the TR-DGU and registered as project number 2016-012.

### Patients

For the development set, surviving patients documented in TR-DGU were selected from a 5 year period (January 2014–December 2018). Only cases admitted to a German trauma center and treated on an intensive care unit (ICU) were considered. Patients with minor injuries defined as Maximum Abbreviated Injury Scale (MAIS) severity grade one were excluded. Primary admitted cases who were not transferred out within 48 h, as well as cases transferred in from other hospitals were considered. This left 109,793 survivors from 670 hospitals for analysis. Before excluding the non-survivors from the development set, mortality rate was 9.7% in those admitted to ICU, and another 1.6% died before admission to ICU.

Patients were further excluded due to the following reasons: Length of stay on ICU not documented (*n* = 6); late transfer in from another hospital with >3 days between accident and transfer (n = 563); transferred out before day 30 in a condition that still required intensive care (intensive care treatment not terminated; *n* = 1,063). After these exclusions data of 108,178 patients were available.

The results of this analysis were validated in a second set of patients documented in TR-DGU from 2019 to 2022, using the same inclusion and exclusion criteria.

Data collection system of TR-DGU allows to document length of intensive care in days or hours. If ICU stay was documented in hours, the respective days were calculated as a decimal number, and parts of a day were counted as a separate day. So, all LoS ranging from 1 to 24 h were counted as 1 day, and 25 h then counted as 2 days, and so on.

Organ failure was documented as Sequential Organ Failure Assessment (SOFA) score grade 3 or 4 for five organ systems: lung/respiration, coagulation, heart/blood pressure, liver, kidney, and the central nervous system (8). Organ failure and sepsis were documented as binary variable (yes/no) during the ICU stay.

For number of injuries, only injuries with an AIS severity level of 2 or more were counted. Often, AIS 1 injuries were not completely documented, and their impact on length of stay could be neglected. Furthermore, the number of injuries were truncated at 13. Only 1.9% of cases had more than 13 diagnoses documented (maximum 29), but without a further effect on LoS.

### Statistics

**Figure 1:**
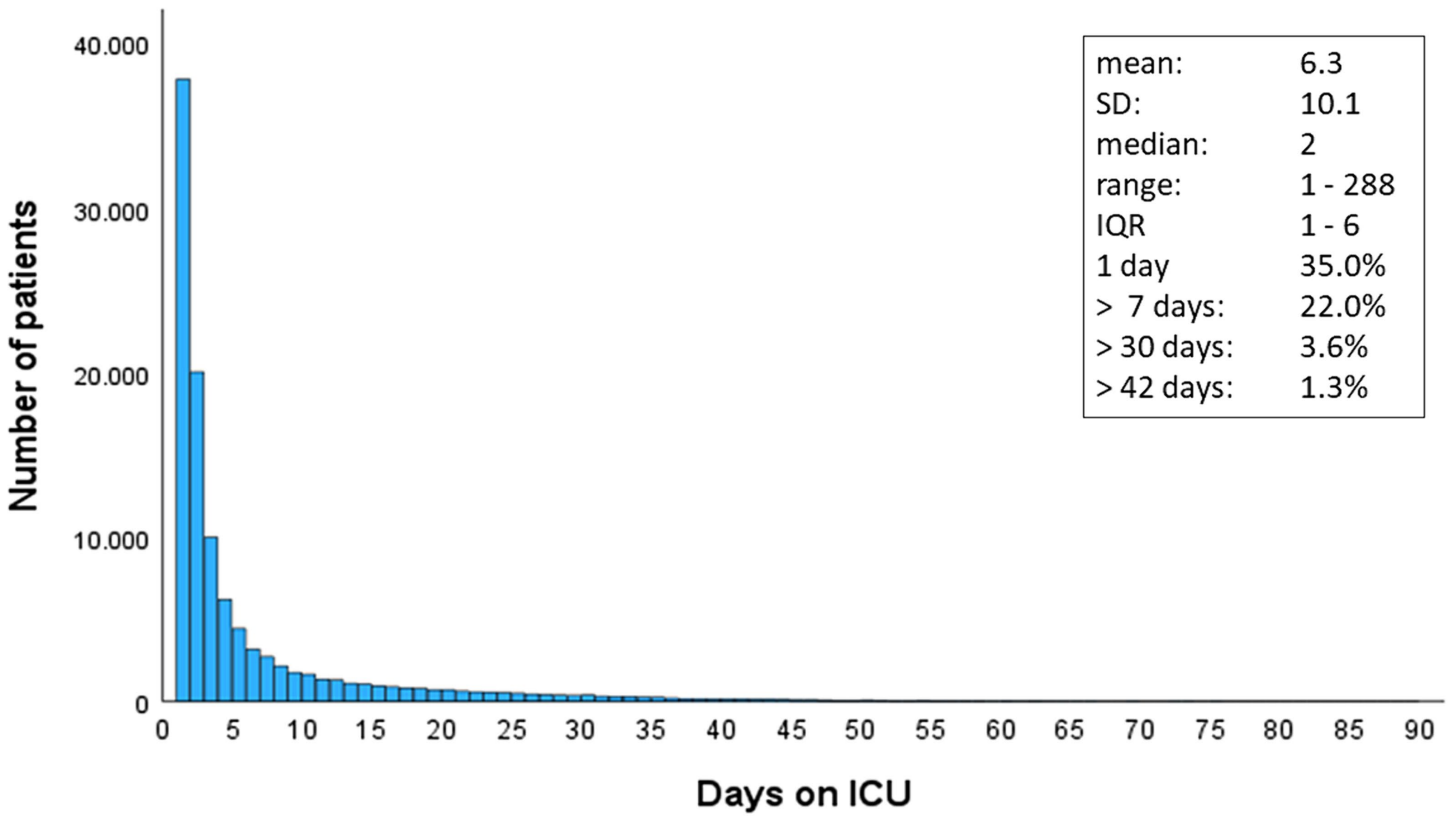
Distribution of length of stay in ICU in 108,178 surviving patients with severe injuries. SD, standard deviation; IQR, inter-quartile range.

Since the distribution of LoS data were heavily skewed with a long tail to the right (Figure 1), standard regression analysis would violate the requirements of this method. Therefore, we followed an approach previously used and published by Kramer and Zimmerman (6). According to their approach, we first defined a threshold for prolonged intensive care. We decided to use a cut-off of 7 days, which means that an ICU stay lasting longer than 1 week (8 days or more) was considered as a prolonged ICU stay. This cut-off was chosen both for clinical and methodological reasons. Short ICU stays often depend more on the availability of beds than on the clinical condition. Furthermore, when including a large number of short ICU stays in a model, then the regression algorithm aims to fit these short stays rather than identifying reasons for a prolonged stay.

After 1 week nearly 80% of patients had left the ICU already. We then considered factors available before first ICU admission to estimate the probability of a prolonged ICU stay using logistic regression analysis. This analysis was performed on the total population of 108,178 cases. Based on the coefficients of the model, a formula was provided to calculate the probability of requiring a prolonged ICU stay. Predicted and observed values were compared, and discrimination was assessed by the area under the receiver operating characteristic (ROC) curve.

In a second step we only used cases with a prolonged ICU stay and tried to predict their length of stay. This analysis would allow including all information until day 7. Similar to the approach of Kramer & Zimmerman, we truncated rather long ICU stays at day 30 for this analysis. This linear regression analysis with a truncated LoS (range 7–30 days) was calculated on 23,830 cases.

Odds Ratios (OR) from logistic regression analysis as well as coefficients from linear regression analysis were presented with their respective 95% confidence intervals.

Counts were presented as percentage, and continuous measures were presented as mean with standard deviation (SD), or as median with quartiles in case of skewed distributions. For observed vs. predicted length of stay, the mean absolute error (MAE) and the root mean squared error (RMSE) were reported. All analyses were performed using SPSS statistical software (version 29, IBM Inc., Armonk NY, United States).

## Results

A total of 108,178 severely injured survivors who required intensive care were documented in TR-DGU within a 5 years period. The mean age was 50 years, and 70% of patients were males (Table 1). The average Injury Severity Score (ISS) was 16.2 points. Many patients required a short ICU stay only (Figure 1); the median LoS was 2 (IQR 1–6) days. Thirty-five percent were discharged within 24 h.

**Table 1:** Basic data and potential predictors for a prolonged need for intensive care (>7 days) in all patients.

	Short	Prolonged	Total
	ICU stay	ICU stay	
Patients	*N* = 84,348	*N* = 23,830	*N* = 108,178
Transfer in from other hospital	6.4%	12.7%	7.8%
Age (years)	49.3 (22.1)	52.8 (21.2)	50.0 (21.9)
Male sex	69.7%	72.9%	70.4%
Injury Severity Score (ISS)	13.6 (7.8)	25.5 (11.6)	16.2 (10.1)
Serious head injury (AIS 3+)	21.0%	49.4%	27.2%
Serious thoracic injury (AIS 3+)	30.4%	50.8%	34.9%
Serious abdominal injury (AIS 3+)	4.8%	12.4%	6.5%
Serious injury of the spinal cord (AIS 3+)	5.6%	13.1%	7.2%
Serious injury of the extremities (AIS 3+)	18.9%	31.9%	21.7%
Penetrating trauma	3.9%	3.3%	3.7%
Number of injuries	3 (2–5)	6 (4–8)	4 (3–6)
Pre-injury status			
ASA 1	56.7%	44.6%	54.1%
ASA 2	29.1%	34.4%	30.2%
ASA 3/4	14.2%	21.0%	15.7%
Blood transfusion before ICU admission	2.4%	15.7%	5.4%
Shock with BP ≤ 90 mmHg prehospital or on admission	3.0%	12.7%	5.1%
Intubated/ventilated on ICU	15.5%	79.3%	29.5%
Sepsis	0.5%	16.4%	4.4%
Multiple organ failure	2.9%	40.7%	13.0%
OF Lung/respiration	2.3%	31.8%	10.1%
OF heart/blood pressure	5.0%	43.5%	15.3%
OF coagulation	2.3%	15.0%	5.7%
OF liver	0.1%	2.0%	0.6%
OF kidney	0.6%	5.8%	2.0%
OF central nervous system	3.3%	32.2%	11.0%

OF, organ failure; ICU, intensive care unit; ASA, American Society of Anaesthesiologists classification; BP, (systolic) blood pressure.

### Prediction of prolonged ICU stay

A prolonged ICU stay of more than 7 days was observed in 23,830 patients (22.0%). These patients differed in many aspects from those with a shorter ICU stay (Table 1). They had about twice as many injuries, and their ISS nearly doubled (13.6 vs. 25.5 points). Mechanical ventilation was observed in 79.3% of cases with a prolonged ICU stay, as compared to only 15.5% in cases with a shorter stay.

Patients with a prolonged ICU stay were responsible for 71.5% of all ICU days, and for 92.7% of all ventilation days.

**Figure 2:**
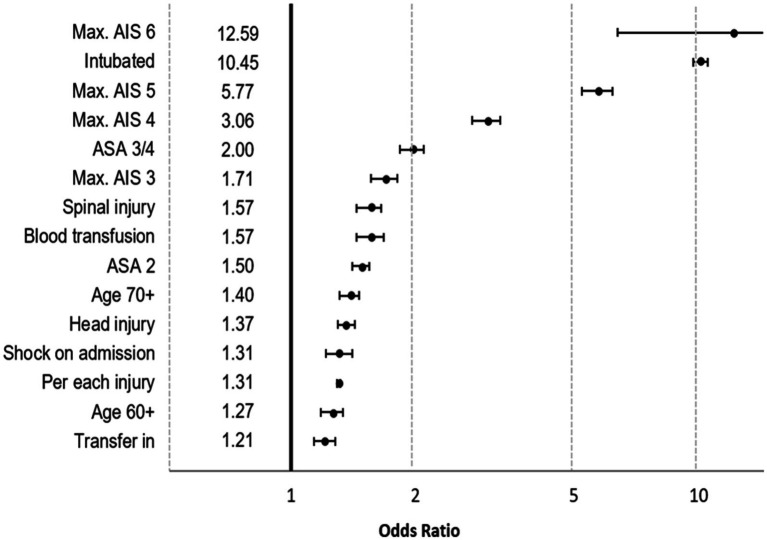
Results of logistic regression analysis for prediction of prolonged ICU stay. Effects are presented as odds ratios with 95% confidence intervals.

Logistic regression analysis was used to develop a prediction model for prolonged ICU stay. The following measures were considered as potential predictors: age; sex; pre-injury status (according to the American Society of Anaesthesiologists (ASA) classification); number of injuries; transfer in from another hospital within 3 days; worst injury severity level (AIS); relevant injury (AIS 3+) in the following body regions: head, thorax, abdomen, spine, and extremities; shock (systolic blood pressure ≤ 90 mmHg pre-hospital or on admission); need for blood transfusion before ICU admission; and need for ventilation on admission to ICU. The number of injuries was the only continuous predictor in the model. The reference category of a categorical predictor was selected based on the lowest risk category so that the remaining categories received an OR above 1.00. The following variables were eliminated from the model due to a minor impact (OR < 1.20): sex and relevant injury of the thorax, the abdomen, and the extremities. The remaining predictors are presented in Figure 2, the full model is given in Supplementary Table 2A. Nagelkerke’s R^2^ of this model was 0.541.

**Figure 3:**
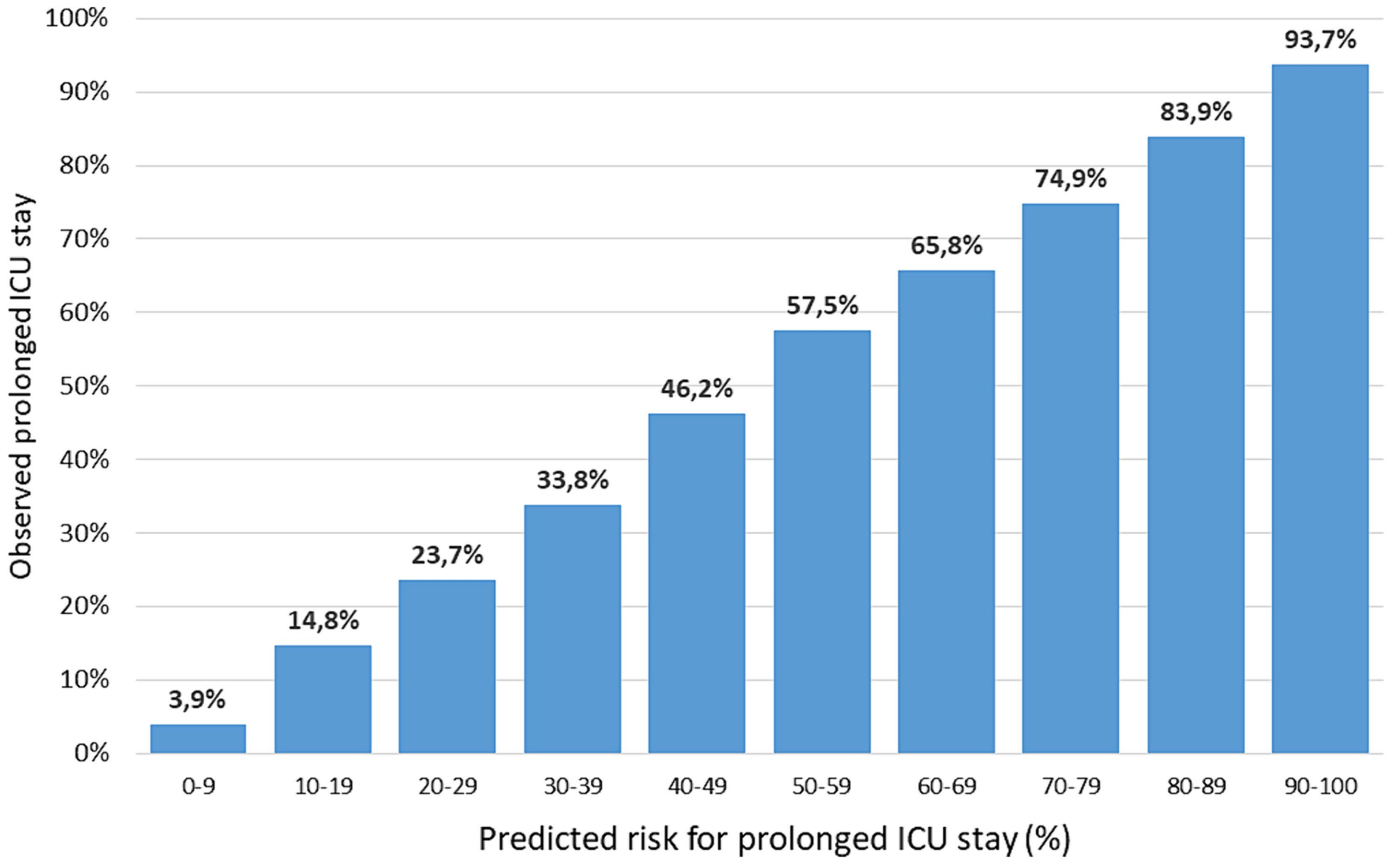
Observed (vertical axis) and predicted (horizontal axis) risk of prolonged ICU stay in the development set. Predicted risk is grouped in 10 categories of equal range; sample sizes per category range from 2,892 (90–100) to 63,011 (0–9).

The most important predictors were need for ventilation (OR 10.45, CI_95_ 10.03–10.89) and survivors with MAIS 6 (OR 12.56, CI_95_ 6.49–24.43; *n* = 81; mostly high cervical spine injury). Based on the coefficients of the model, a formula was derived for calculation of the risk for prolonged ICU stay (Table 2). According to this formula, 21.9% of patients were expected to need a prolonged ICU stay. Figure 3 compares observed and predicted risk for a prolonged ICU stay in patients with different risk levels. The majority of patients (58.2%) had a low probability <10% for a prolonged ICU stay.

**Table 2:** Formula for calculating the risk of prolonged ICU stay (8 or more days).

Predictor	Reference	Points weights	
Constant	---	**−4.83**	
Age	<60 years	**+0.24**	if 60–69 years old
		**+0.33**	if 70 years or older
Number of injuries	---	**+0.27**	per each injury (max. 13)
Worst injury	AIS 2	**+0.54**	if AIS = 3
		**+1.12**	if AIS = 4
		**+1.75**	if AIS = 5
		**+2.53**	if AIS = 6
Head injury	AIS 0–2	**+0.32**	if AIS 3–6
Spinal injury	AIS 0–2	**+0.45**	if AIS 3–6
Ventilation on ICU	No	**+2.35**	if ventilated
Pre-injury status	ASA 1	**+0.40**	if ASA = 2
		**+0.69**	if ASA = 3 or 4
Blood transfusion before ICU admission	no	**+0.45**	if yes
Shock pre-clinical or on admission	no	**+0.27**	if yes
Transfer in from other hospital	no	**+0.19**	if yes
Let **X** be the sum of point weights per case. Using the exponential function with Euler’s number ***e***, the risk of prolonged ICU stay is then calculated as: RISK = ***e***^**X**^/(1 + ***e***^**X**^).			

For values in the reference category, no points were added. The point weights were derived from the coefficients of the logistic regression analysis (full model: see Supplementary Table 2A).Bold values are used in the formula for calculating the risk of prolonged ICU stay.

### Length of stay prediction

In a second step only survivors with a prolonged ICU stay were considered (at least 8 days; *n* = 22,830). Patients with a very prolonged ICU stay (*n* = 3,906; 16.4%) were not excluded but their LoS was truncated at 30 days for this analysis. The linear regression analysis used the same predictors as in the first step, plus the information whether a case was still intubated and ventilated at day 8.

**Figure 4:**
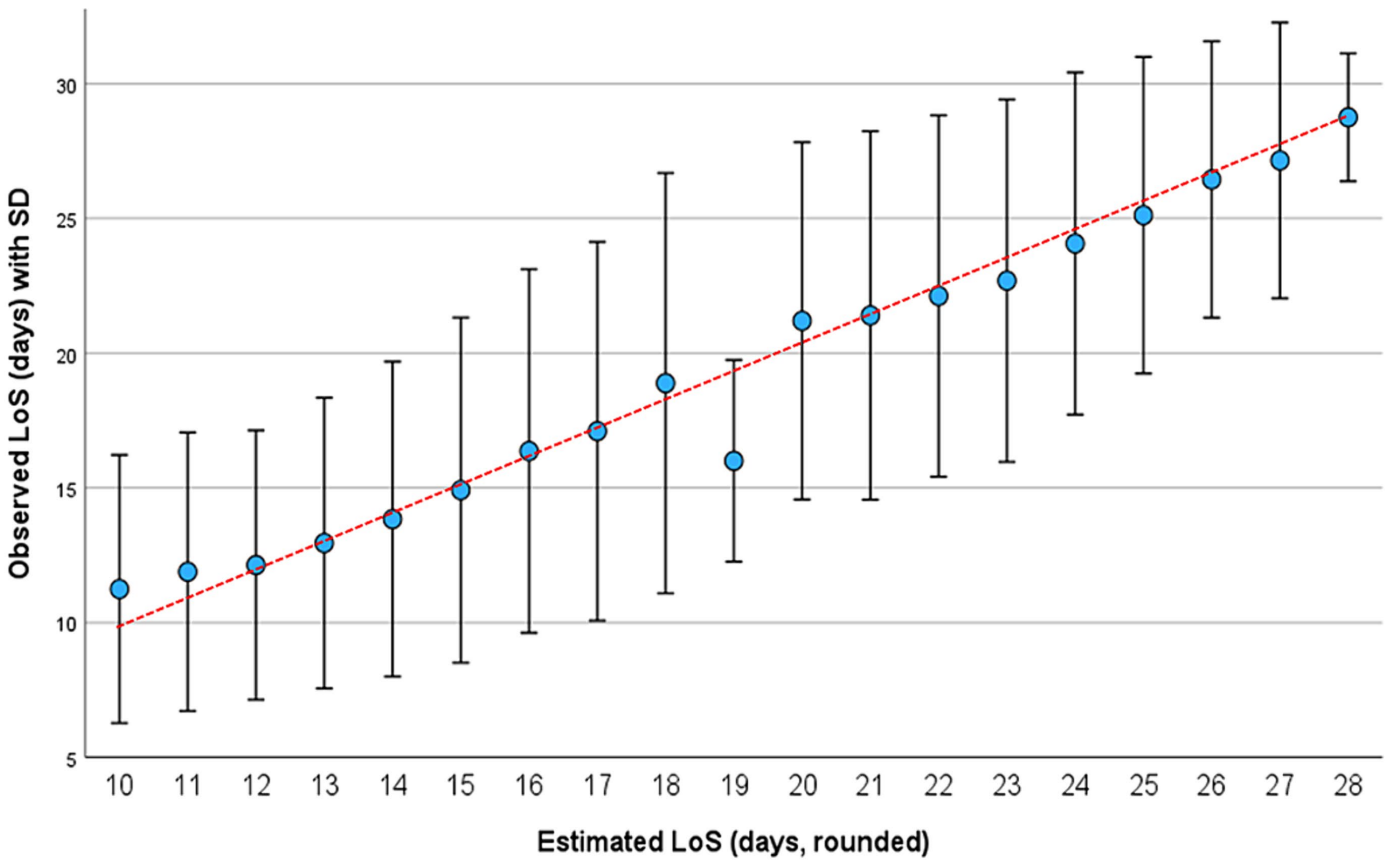
Observed (vertical axis) and estimated (horizontal axis) LoS on ICU in 23,830 patients of the validation set. Long stays were truncated at 30 days. Results are presented as mean with standard deviation (SD).

All predictors, except for “transfer in,” had an effect size of at least 0.2 days on LoS and were included in the final model. Number of injuries and severity level of the worst injury (max AIS) had a linear effect on LoS, but age did not. Table 3 describes the final linear model where the coefficients correspond to days. The R^2^ was 0.40 so that nearly half of the variation could be explained by the model. Table 4 gives the final formula for calculating the expected number of days on ICU. Starting with the constant term (9.4 days) values of 0.2 to 8.8 were added in case of the respective finding. Requiring ventilation beyond day 7 is by far the strongest predictor (+8.8 days), and only thoracic trauma reduces the estimated LoS.

**Table 3:** Results of linear regression analysis of length of stay on ICU for 23,830 patients with a prolonged ICU stay (8–30 days).

Parameter	Prevalence	Coefficient	95% CI
Age ≥ 50 years	57.2%	0.49	0.30–0.68
Male sex	72.9%	0.56	0.39–0.74
Pre-injury status			
ASA 2	33.5%	0.61	0.41–0.81
ASA 3 or 4	21.0%	1.09	0.85–1.33
Maximum AIS severity (2–6)^*^	---	0.21	0.11–0.31
Number of injuries (1–13)^*^	---	0.26	0.22–0.29
Serious head injury	49.4%	0.40	0.21–0.60
Serious thoracic injury	50.8%	−0.57	−0.75–−0.38
Serious abdominal injury	12.4%	0.51	0.26–0.76
Serious spinal injury	13.1%	0.84	0.60–1.09
Serious extremity injury	31.9%	0.77	0.57–0.96
Blood transfusion	15.7%	0.97	0.73–1.20
Shock	12.7%	0.44	0.20–0.69
Ventilated on ICU	79.3%	0,65	0.43–0.88
Ventilated > 7 days on ICU	47.0%	8.82	8.64–9.00

^*^Variable with multiple values; coefficient is the effect per one point.

**Table 4:** Formula for calculating the estimated number of days on ICU in patients with prolonged ICU stay (8–30 days).

**Estimated ICU LoS** =	9.4 days	(constant)
	+0.5 days	if age ≥ 50 years
	+0.6 days	if male sex
	+0.6 days	if pre-injury status was ASA 2
	+1.0 days	if pre-injury status was ASA 3/4
	+0.25 days	multiplied with number of injuries (1–13)
	+0.2 days	multiplied with maximum AIS severity (2–6)
	+0.4 days	if serious head injury (AIS 3+)
	–0.6 days	if serious thoracic injury (AIS 3+)
	+0.5 days	if serious abdominal injury (AIS 3+)
	+0.8 days	if serious spinal injury (AIS 3+)
	+0.8 days	if serious injury of the extremities (AIS 3+)
	+1.0 days	if blood transfusion before ICU admission
	+0.4 days	if shock preclinical or on admission
	0.7 days	if ventilated on ICU
	+8.8 days	if still ventilated on day 7

The observed length of stay in this group was 18.0 days (SD 7.8; median 16; IQR 11–25; range 8–30). The mean value for the estimated length of stay was 17.9 days (SD 15.6; median 15.6; IQR 13–23; range 9.9–28.0). Observed and predicted values were highly correlated (r = 0.633) (Figure 4). The mean absolute error (MAE) was 5.0 days, and the root mean squared error (RMSE) was 6.1 days.

### Validation

**Figure 5:**
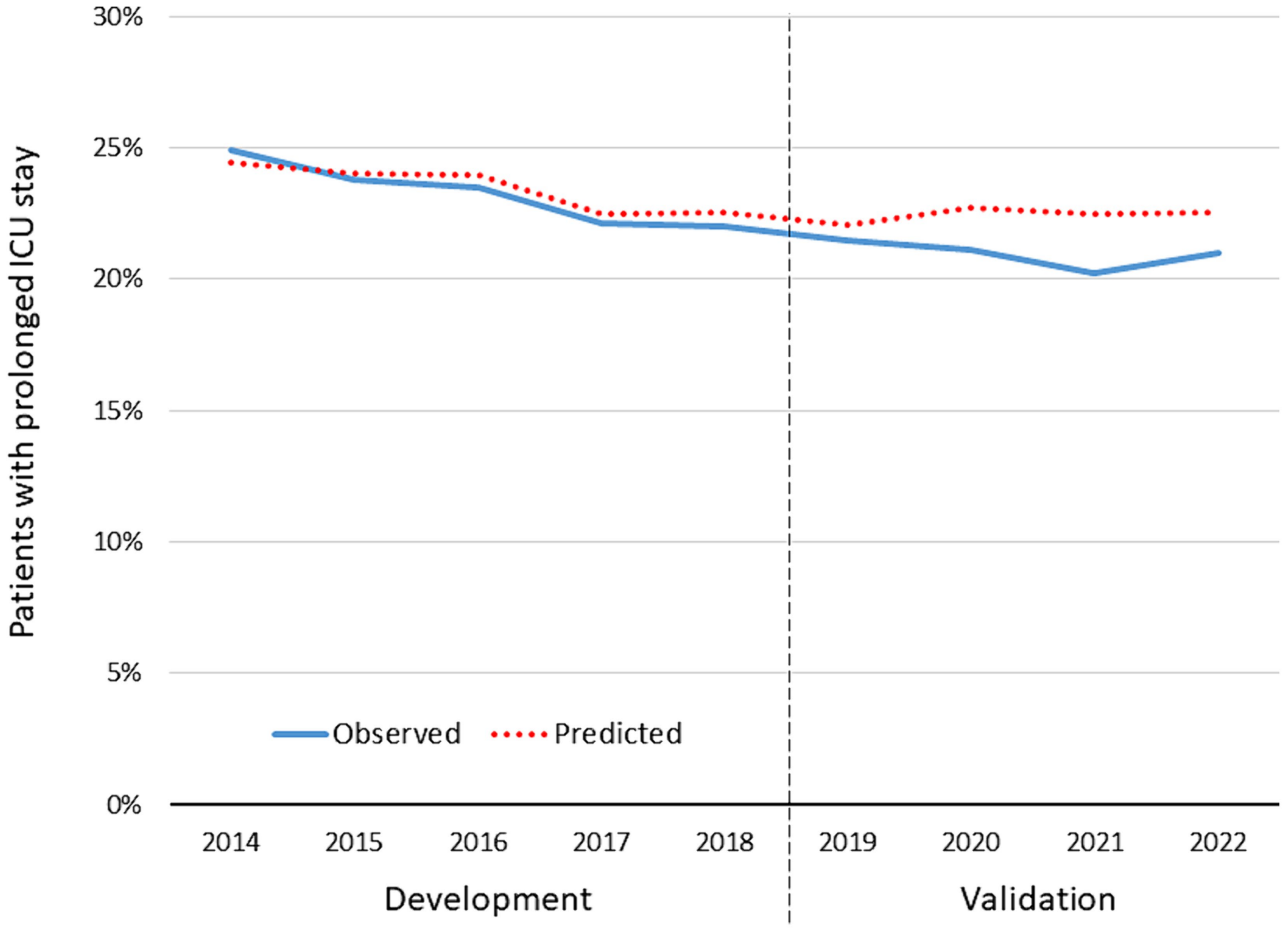
Observed and expected rate of patients with a prolonged ICU stay in survivors treated on ICU (*n* = 180,240). The years 2014–2018 served as development data for the prediction model; the years 2019–2022 served as validation for the model.

**Figure 6:**
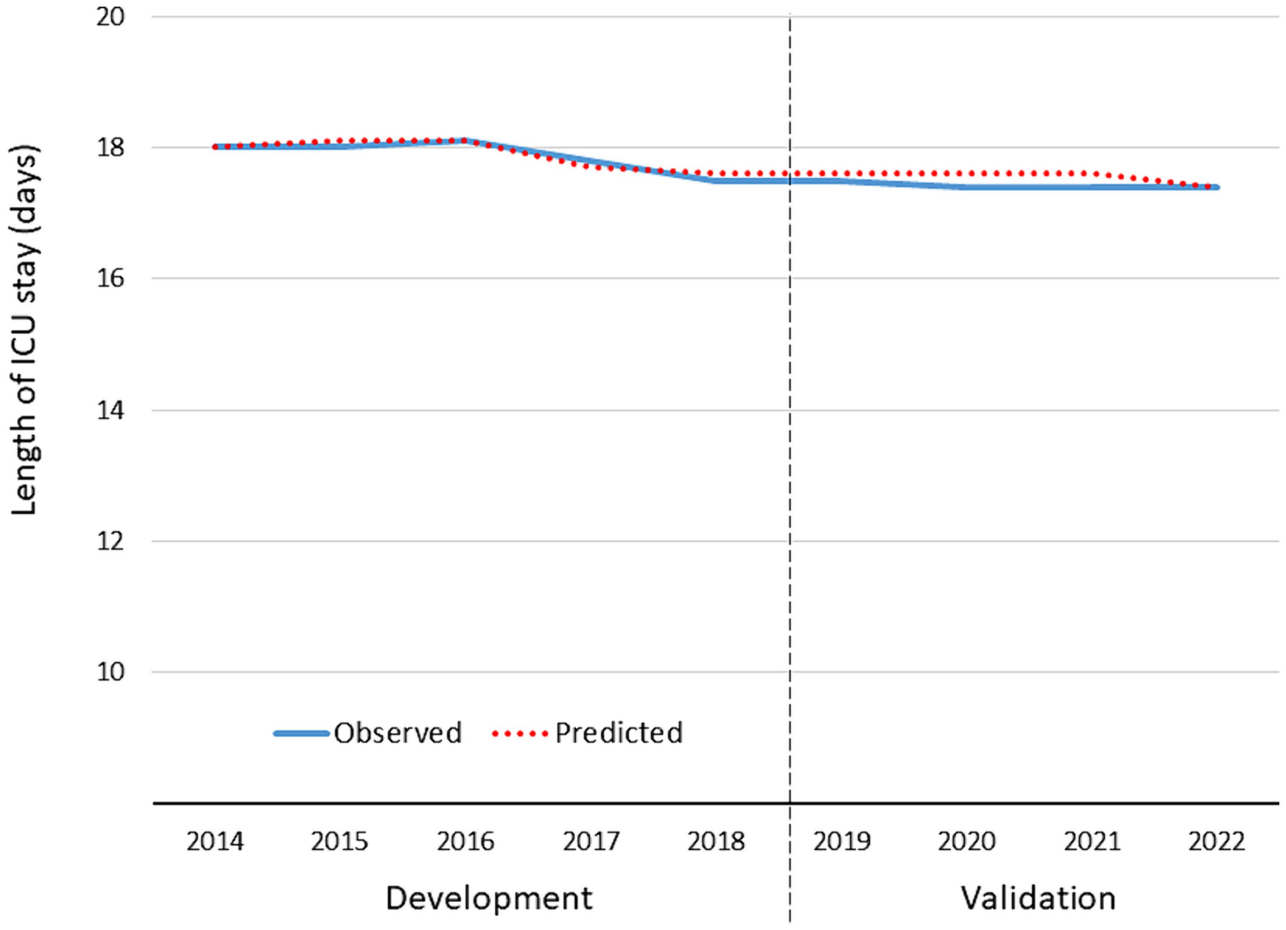
Observed and expected average length of ICU stay in patients with a prolonged ICU stay > 7 days (*n* = 38,064). The years 2014–2018 served as development data for the prediction model; the years 2019–2022 served as validation for the model.

Using the same inclusion and exclusion criteria, a total of 72,062 patients from TR-DGU (2019–2022) were available for validating the previous results. Table 5 summarizes the results. Figures 5, 6 present observed and expected values per year for both measures, risk of requiring a prolonged ICU stay and expected LoS in patients with a prolonged stay.

**Table 5:** Summary results from the development and validation dataset.

	Development dataset	Validation dataset
Years	2014–2018	2019–2022
Number of patients	*n* = 108,178	*n* = 72,062
ICU length of stay (days)^*^	2 (1–6)	2 (1–6)
	6.3 days	5.7 days
Prolonged ICU stay (>7 days)	*n* = 23,830	*n* = 14,243
	22.0%	19.8%
Expected rate of patients with a prolonged ICU stay	21.9%	20.8%
Area under the ROC curve, with 95% confidence interval	0.903 (0.900–0.905)	0.895 (0.892–0.898)
**For patients with prolonged ICU stay**		
Length of stay (range 8–30)^*^	16 (11–25)	15 (10–25)
	18.0	17.5
Estimated length of stay (days)^*^	15.6 (13.3–22.8)	14.8 (13.2–22.6)
	17.9	17.5
Pearson’s correlation of observed and predicted length of stay	0.613	0.611

^*^Median with IQR, mean.

## Discussion

The aim of this project was to establish a benchmark for surviving patients in external quality control. While adjusted risk of death prediction is established in nearly all trauma registries, only few outcome indicators are available for survivors. Besides quality of life assessment (which is hard to implement), complication rates and length of stay are candidates (9). We agree with Kramer who stated in a recent review that “ICU LoS predictions should not be used for individual patients, but can be useful for benchmarking efficiency across ICUs and patient groups” (6). Both measures, however, require an adjustment for injury severity, like mortality. Excellent hospitals that could prevent severe cases from dying will have more organ failure and longer intensive care in survivors.

Previous attempts to measure resource use include the Standardized Resource Use (SRU) quantification published by Rothen et al. (10) which is plotted against the standardized mortality rate (SMR). This approach has been developed in a general ICU population including all patients, also non-survivors. It estimates expected ICU LoS per survivor in different severity strata, and cumulated expected number of days were compared to observed ones. However, this approach also distributes resources used in non-survivors among the surviving patients, and therefore SRU is strongly correlated to mortality and the SMR. The present approach is limited to survivors only, and it uses LoS predictors specifically available for severe trauma patients only.

Prediction of length of stay is a methodological challenge since data are rather skewed. Several methods were suggested in the literature where each methods has its strengths and weaknesses (7, 11, 12). We applied the approach of Kramer et al. which has some appealing properties: The first step is a classical binary prediction model for a prolonged ICU stay. In a second step, LoS will be predicted in those patients with a prolonged stay only. This means that for the large number of patients with a limited need for intensive care no LoS prediction will be performed. It has been reported that including also patients with a short ICU stay will lead to a model that very much focuses on these short stay patients, and prediction of longer stays become uncertain (13, 14).

Also most recently published LoS prediction models like the one by Peres et al. perform a parallel prediction of the risk of a long stay (15). But like other prediction models, their intention is an early identification of long stay patients, including non-survivors, in a general intensive care unit.

In our study 78% of patients left ICU within 1 week. This cut-off value was chosen based on clinical reasoning since major complications like sepsis or multiple organ failure usually would require more than 1 week of intensive care. In short stay patients, it is less important whether LoS was 2, 3, or 4 days. Such a decision often depends on organizational or other reasons rather than solely on the patient’s condition. So the first step is calculated in all cases, and the focus is on needing a substantial amount of intensive care.

Only in a second step LoS is directly predicted using a regression analysis. As previously recommended, very long stays were truncated at 30 days (14, 16). In our study only 3.6% of cases had a stay of more than 30 days. Among these cases with a very long ICU stay only one third required a stay longer than 42 days. But those cases would seriously influence the prediction model.

Our first model was able to identify several predictors for a prolonged intensive care in survivors. As expected, the severity (here: worst AIS severity level) and the number of injuries are the strongest predictors, combined with the need for mechanical ventilation in ICU. Also pre-existing diseases (pre-injury ASA status) and higher age predict a prolonged ICU stay. Among specific injuries, spinal cord and head injuries were relevant predictors while injuries to the rest of the body only showed a marginal additional effect, after adjustment for severity. The final model was able to explain a lot of the observed variation (Nagelkerke’s R^2^ = 0.54). The validation of this model in the years 2019–2022 showed good results, with only 1.0% difference between observed and predicted rates. This difference is mainly based on the most recent 2 years where less patients needed a prolonged ICU stay. This might be the continuation of a previously published trend of shorter ICU length of stay observed by Böhmer et al. (17) in the same registry. It might reflect improvements in intensive care in the early care, but this is speculative.

Moore et al. also tried to predict LoS on ICU in severe trauma patients (9). They found that injury severity (worst AIS in six body regions) and age each contributed more than one third of the explained variation of the model. The remaining predictors (comorbidities, mechanism of injury, transfer, GCS, repeated ICU visit) together explained the rest. This is similar to risk of death prediction where injury severity and age also are the most important predictors. The prediction model of Kramer and Zimmerman who also predicted a prolonged ICU stay first (more than 5 days) used a general ICU population (6). They also found that the need for ventilation on day one was highly predictive. They found an even higher effect for “unable to assess GCS” which is obviously associated with sedation and mechanical ventilation. Since trauma patients were just a small subgroup in the data of Kramer and Zimmerman, they used the general Acute Physiology Score [from APACHE IV (14)] instead of injury severity. Other authors used similar general severity scores, like the Simplified Acute Physiology Score (SAPS II) or the Mortality Prediction Model (MPM) (18, 19).

The second model is a linear regression predicting length of stay, truncated at 30 days, in patients with prolonged ICU stay. We used real days here, and not a transform of LoS, so that the coefficients could directly be interpreted as number of days in the final model. There is one exceptional factor among the predictors, which is need for a prolonged need for artificial ventilation on day 8. This finding would add 8.8 days to the constant value of 9.4 days. None of the other predictors had a similar effect size. The only predictor with a negative weight was thoracic trauma (−0.6 days).

The outstanding importance of artificial ventilation for LoS prediction has also been found in previous analyses. Peres et al., for example, found ventilation to be the most important factor, applying various machine-learning approaches (15).

Validation of predicted LoS showed a nearly perfect concordance with observed LoS. During the validation phase LoS was about 0.5 days shorter than in the development phase. This corresponds with the slightly lower risk for a prolonged stay observed in the first model.

Future studies in this area will focus on further validation analyses with existing LoS prediction models, like Standardized Resource Use (SRU) (10) and their applicability in the subset of trauma ICU patients. The Standardized Length of Stay Ratio (SLOSR) approach showed already improved results compared to SRU (20). The focus on survivors only, as we did here, does not require to limit resources in non-survivors. Thus long ICU stays in patients who finally died will not affect the LoS estimation. Our approach could serve as a perfect complement to severity-adjusted mortality prediction, and the combination of both seems promising [as Rothen et al. did (10)]. Further analyses will focus on early complications in patients with less than 7 days on ICU.

### Limitations

Length of stay prediction is not an easy task, as mentioned above. The approach which we used may not be the best strategy. Other methods including transformations, machine learning, or different regression models may have reached superior results. However, the observed R^2^ values were large enough to support an application in benchmarking. Furthermore, the formulas we derived for calculating expected need for a prolonged ICU stay, as well as LoS, are based on coefficients rounded to one decimal. This seemed to be reasonable regarding the respective confidence intervals. Furthermore, using days instead of some transformation thereof might be suboptimal, but on the other hand, the results could directly be interpreted as days. This is an advantage when communicating the results to clinicians.

The validation period coincides with the COVID pandemic. During that phase, intensive care has been challenged a lot, and therapeutic changes may have occurred. However, separate analyses from the TR-DGU did not suggest that less trauma patients received intensive care, nor that length of stay did change during the pandemic (21).

As has been observed in the past, intensive care is rather resource-consuming, and economic challenges may have future impact on ICU length of stay. Thus a continuing re-validation and potential calibrations are mandatory.

Finally, such models will not necessarily reflect the situations in other countries. The results may depend on the availability of ICU beds, and on how intensive care is refunded.

## Conclusion

Length of stay on ICU is an adequate outcome measure in survivors after severe trauma and could be used in benchmarking after adjustment for severity. We developed a prediction model for a prolonged ICU stay (>7 days), and an estimator for LoS in patients who needed intensive care for more than 1 week. Both instruments were validated and will be used in future quality reports.

## Data availability statement

The data analyzed in this study is subject to the following licenses/restrictions: according to the guidelines of the German Trauma Society (DGU) and the Akademie der Unfallchirurgie (AUC GmbH) who runs the registry, data from TR-DGU are not publicly available. However, applications for analyses could be forwarded to AUC. The corresponding author will answer requests for data details regarding the present project. Requests to access these datasets should be directed to support-tr@auc-online.de.

## Ethics statement

The present study is exclusively based on routinely collected data gathered for the purpose of legally required external quality control. Thus no patient consent was required. Our responsible ethics committee (University Witten/Herdecke, Germany) decided regarding a previous similar request (reference no. 64/2018) that no separate vote is required when such de-personalized data are analyzed.

## Author contributions

RL: Formal analysis, Methodology, Validation, Visualization, Writing – original draft, Writing – review & editing. CW: Conceptualization, Supervision, Validation, Writing – original draft, Writing – review & editing.
