# Supplementary material for: Prediction of prolonged length of stay on the intensive care unit in severely injured patients—a registry-based multivariable analysis
Source: Front Med (Lausanne). 2024 Jun 5;11:1358205. doi: 10.3389/fmed.2024.1358205 (PMC11188296; doi:10.3389/fmed.2024.1358205)
Supplement: Supplementary file 1 [file Data_Sheet_1.PDF]

Supplementary Table 2a

Results from logistic regression analysis with prolonged ICU stay (8 or more days) as dependent vatiable. Nagelkerke's $R^2 = 0.541$

**Predictor**	**Value**	**Coefficient**	**SE**	**Odds Ratio**	**95% CI**	**p-value**
Age (Ref: <60)	60-69	0.236	0.032	1.27	1.19 – 1.35	<.001
	70+	0.334	0.030	1.39	1.32 – 1.48	<.001
Number of injuries	per injury	0.272	0.005	1.31	1.30 – 1.33	<.001
AIS severity of worst injury (Ref: 2)	3	0.535	0.039	1.71	1.58 – 1.84	<.001
	4	1.120	0.041	3.06	2.83 – 3.32	<.001
	5	1.752	0.045	5.77	5.28 – 6.30	<.001
	6	2.533	0.338	12.59	6.49 – 24.43	<.001
Head injury AIS 3+ (Ref: no)	yes	0.318	0.023	1.37	1.32 – 1.44	<.001
Spinal injury AIS 3+ (Ref: no)	yes	0.450	0.035	1.57	1.46 – 1.68	<.001
Ventilation on ICU (Ref: no)	yes	2.347	0.021	10.45	10.03 – 10.89	<.001
ASA pre-injury status (Ref: 1)	2	0.403	0.026	1.50	1.42 – 1.57	<.001
	3/4	0.693	0.034	2.00	1.87 – 2.14	<.001
Blood transfusion before ICU (Ref: no)	yes	0.452	0.039	1.57	1.46 – 1.70	<.001
Shock, pre-hospital or on admission; syst. BP <= 90 mmHg (Ref: no)	yes	0.272	0.039	1.31	1.22 – 1.42	<.001
Transfer in from other hospital (Ref: no)	yes	0.194	0.032	1.21	1.14 – 1.29	<.001
Constant		-4.825	0.039			<.001

Ref = reference category; CI = confidence interval; SE = standard error; BP = blood pressure; ICU = intensive care unit; AIS = Abbreviated Injury Scale
