# Supplementary figures and images for: Prediction of prolonged length of stay on the intensive care unit in severely injured patients—a registry-based multivariable analysis
Source: Front Med (Lausanne). 2024 Jun 5;11:1358205. doi: 10.3389/fmed.2024.1358205 (PMC11188296; doi:10.3389/fmed.2024.1358205)

### **Supplemental Figure 1**

Flow chart of included and excluded patients.

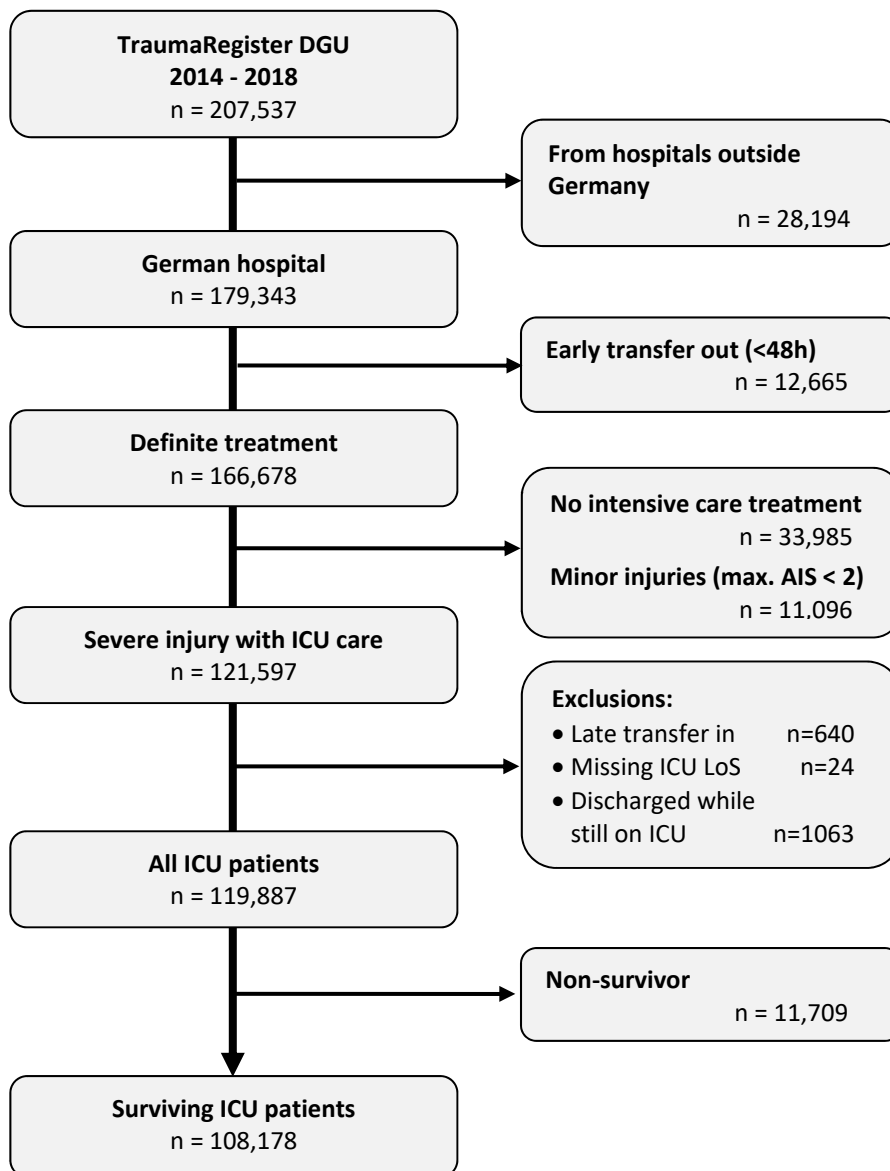

Supplement: Supplementary file 2 [file Data_Sheet_2.PDF]
